# Breve actualización sobre el diagnóstico de la enfermedad por coronavirus 2019 (COVID-19)

**DOI:** 10.1515/almed-2020-0103

**Published:** 2020-11-13

**Authors:** Giuseppe Lippi

**Affiliations:** Sección de Bioquímica Clínica, Hospital Universitario de Verona, Piazzale L.A. Scuro, 10,37134 Verona, Italy, Phone: 0039-045-8122970, Fax: 0039-045-8124308,

**Keywords:** coronavirus, COVID-19, diagnóstico, virus

La enfermedad por coronavirus 2019 (COVID-19), causada por un virus llamado SARS-CoV-2 (coronavirus tipo 2 del síndrome respiratorio agudo grave) ya ha afectado a varios millones de personas en todo el mundo, habiendo causado más de un millón de muertes. El SARS-CoV-2 es un coronavirus típico perteneciente a la misma familia de virus que causan el resfriado común y los que provocaron los dos brotes anteriores de síndrome respiratorio agudo (SARS) en China entre 2002 y 2003, y el síndrome respiratorio de Oriente Medio (MERS) de 2012 [1]. Al igual que otros miembros de la familia del coronavirus, el SARS-CoV-2 está formado por una sola cadena de ARN positivo (∼30.000 nucleótidos), que codifican proteínas tanto estructurales como funcionales. Entre las proteínas estructurales se incluyen las llamadas glicoproteínas de espiga (S), las proteínas de envoltura (E), las proteínas de membrana (M) y las proteínas de la nucleocápside (N), que rodean el genoma del virus. La proteína S media la unión del virus a las células anfitrionas, permitiendo al SARS-CoV-2 penetrar en la célula y realizar su actividad citotóxica intracelular. Entre las proteínas funcionales más importantes se encuentra la ARN polimerasa dependiente de ARN (RpRP), que es la enzima responsable de la replicación del genoma viral en las células anfitrionas [1].

A diferencia de los coronavirus comunes, responsables de las infecciones del tracto respiratorio superior (que se suelen manifestar en forma de resfriado o síndromes catarrales), el SARS-CoV-2 causa una patología grave que inicialmente afecta al tracto respiratorio inferior (p.ej. con síntomas como la neumonía intersticial bilateral, con posible progresión a síndrome de dificultad respiratoria aguda (SDRA), pudiendo afectar a diversos órganos y tejidos, provocando el desarrollo de un síndrome de fallo multiorgánico, que puede causar la muerte a alrededor del 50% de los pacientes con COVID-19 [1]. Hasta la fecha, la mortalidad estimada de COVID-19 es de alrededor del 3%, lo que es una cifra 30 veces superior a la de la gripe (0,1%) [2].

Aunque la evolución biológica y epidemiológica de esta enfermedad sigue siendo indescifrable y considerablemente impredecible, existe la percepción generalizada de que la humanidad tendrá que aprender a convivir con el SARS-CoV-2 y, por tanto, a manejar esta nueva infección a largo plazo [3]. Ante esta perspectiva, tal como indica el mantra de “tests, tests, tests” repetido constantemente por la Organización Mundial de la salud (OMS), resulta indispensable la adopción de protocolos orientados al diagnóstico temprano, exacto y extendido de las infecciones por SARS-CoV-2, así como en el establecimiento de un manejo terapéutico adecuado y oportuno.

Como ocurre con otras enfermedades infecciosas, el diagnóstico de la infección aguda de COVID-19 se basa en la detección del virus o sus constituyentes (principalmente, material genético y/o proteínas) en muestras biológicas. La OMS define como “caso” de COVID-19 como: “*persona con infección por el virus de la COVID-19 confirmada mediante pruebas de laboratorio, independientemente de los signos y síntomas clínicos*” [4], de manera que existe un amplio abanico de técnicas diagnósticas, con diferentes dianas y tiempo de entrega de resultados [5]. Tal como hemos comentado anteriormente, la diana biológica puede ser material genético o una proteína. Según las recomendaciones de la OMS, la identificación de material genético (esto es, ARN) del SARS-CoV-2 es la estrategia diagnóstica de referencia en el diagnóstico de una infección aguda [4]. Aunque la saliva se postula como una alternativa válida e incluso más cómoda [6], los hisopos nasales y orofaríngeos (recogidos simultáneamente) siguen siendo el material de referencia para la detección de ARN vírico. Esta técnica diagnóstica se define como “molecular”, ya que se basa en la amplificación de los ácidos nucleicos virales (test de amplificación de ácidos nucleicos, NAT) presentes en la muestra. La técnica analítica más extendida se basa en el principio de la reacción en cadena de la polimerasa (RT-PCR, por sus siglas en inglés), que consiste en el empleo de sofisticados instrumentos, con un elevado tiempo de respuesta (entre 3 y 5 horas para obtener los resultados), con un rendimiento modesto, ya que solo permite procesar simultáneamente un número relativamente bajo de muestras biológicas (normalmente menos de 100). Aparte de la RT-PCR, están surgiendo otras técnicas analíticas, como la amplificación isotérmica mediada por bucle de transcripción inversa (RT-LAMP, por sus siglas en inglés), que se van extendiendo [4]. Estos métodos alternativos, aunque presentan limitaciones tales como un bajo rendimiento diagnóstico (p.ej. baja sensibilidad, que se traduce en una menor eficiencia a la hora de identificar cargas virales bajas) y permiten procesar simultáneamente un número reducido de muestras (normalmente <10), también permitirán obtener resultados rápidos (en menos de 45°minutos), siendo así adecuados en aquellas circunstancias en las que se precise un diagnóstico rápido (para el cribado de casos sintomáticos en la sala de urgencias, o para realizarles los tests a sujetos que han estado en otros países o en zonas endémicas de COVID-19) [7].

**Figura 1:**
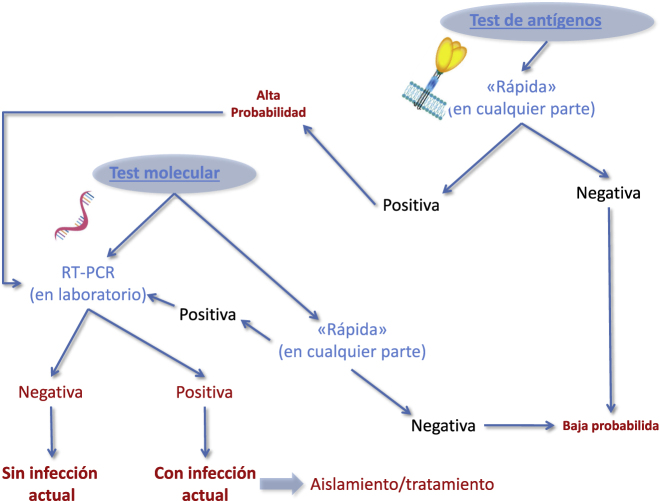
Algoritmo para diagnosticar la infección por coronavirus tipo 2 del síndrome respiratorio agudo grave (SARS-CoV-2), basada en la integración de pruebas moleculares y de antígenos.

Aunque la relevancia clínica de los tests (rápidos) de antígenos está igualando a la de las pruebas moleculares, estas técnicas merecen mención aparte. Según los últimos datos, la sensibilidad de los tests de antígenos validados (existen actualmente multitud de kits en el mercado, algunos de los cuales carecen de un rendimiento diagnóstico aceptable), es menor que la de las pruebas moleculares [7]. En general, los test rápidos de antígenos permiten identificar a los sujetos con una carga viral media o alta de SARS-CoV-2, que son los más contagiosos y los que muestran una evolución clínica menos favorable. Estos tests, basados en la detección de antígenos del SARS-CoV-2, son una técnica de cribado rápida, especialmente en circunstancia en las que se necesita realizar tests rápidos (p.ej. escuelas, centros de trabajo, tras un viaje de larga distancia que implique el paso por un aeropuerto, estación de tren o cruzar la frontera, entre otros). Un resultado negativo podría permitir clasificar al sujeto como “con baja probabilidad” (de infección y/o infectividad), mientras que un resultado positivo deberá ser confirmado mediante RT-PCR, tal como se muestra en la Figura 1. Algunos estudios recientes han permitido estimar que el uso de los tests de antígenos en el cribado podría reducir los costes diagnósticos en más de un 50%, reduciendo también drásticamente el tiempo requerido para confirmar o descartar la enfermedad [8]. Son necesarios más estudios para definir el impacto clínico de estos tests en la segunda ola, especialmente en los sujetos asintomáticos, presintomáticos o levemente sintomáticos con una baja carga viral y/o diseminación de virus viables [9].

Existe una tercera categoría de técnicas diagnósticas, que entra en la definición de “serología” [4]. Este tipo de tests están orientados a detectar la presencia de una respuesta inmune humoral, caracterizada por el desarrollo de inmunoglobulinas (Ig) contra el virus, y cuya relevancia clínica no supera la de la detección de ARN vírico. Este editorial subraya que la eficacia diagnóstica de la serología en la infección por SARS-CoV-2 sigue siendo limitada, ya que la aparición de anticuerpos del SARS-CoV-2 no ocurre en los estadios iniciales (suele producirse entre 5 y 7 días después de haber contraído la infección), ni permite distinguir un contagio reciente de SARS-CoV-2 de una infección anterior [5], [6]. Esto es atribuible a la particular respuesta humoral que se produce contra el virus, dado que un porcentaje variable (entre el 50 y el 90%, principalmente los asintomáticos) de pacientes con COVID-19 no presentan IgM, que suele caracterizar la fase aguda de la mayoría de las infecciones [10]. También se ha señalado que la IgM aparece, como mucho, de forma concomitante con IgG e IgA, aunque frecuentemente aparezce posteriormente. De acuerdo con este sorprendente hallazgo biológico, aunque es poco probable que las pruebas serológicas puedan sustituir a las técnicas moleculares y/o antigénicas, estas jugarán un papel importante en los estudios epidemiológicos y/o en la vigilancia de la salud, a la hora de identificar a los sujetos que han estado en contacto con el virus y han desarrollado una respuesta inmunológica.
